# Despeckling of Ultrasound Images Using Block Matching and SVD in Sparse Representation

**DOI:** 10.3390/s22145113

**Published:** 2022-07-07

**Authors:** Rogelio Reyes-Reyes, Gibran H. Aranda-Bojorges, Beatriz P. Garcia-Salgado, Volodymyr Ponomaryov, Clara Cruz-Ramos, Sergiy Sadovnychiy

**Affiliations:** 1 Instituto Politecnico Nacional, Av. Santa Ana 1000, Mexico City 04440, Mexico; rreyesre@ipn.mx (R.R.-R.); garandab1900@alumno.ipn.mx (G.H.A.-B.); bgarcias1404@alumno.ipn.mx (B.P.G.-S.); ccruzra@ipn.mx (C.C.-R.); 2 Instituto Mexicano del Petroleo, Mexico City 07730, Mexico; ssadovny@imp.mx

**Keywords:** ultrasound sensors, ultrasound image, speckle, superpixel segmentation, block matching, mutual information, singular value decomposition, denoising

## Abstract

This work proposes a novel scheme for speckle suppression on medical images acquired by ultrasound sensors. The proposed method is based on the block matching procedure by using mutual information as a similarity measure in grouping patches in a clustered area, originating a new despeckling method that integrates the statistical properties of an image and its texture for creating 3D groups in the BM3D scheme. For this purpose, the segmentation of ultrasound images is carried out considering superpixels and a variation of the local binary patterns algorithm to improve the performance of the block matching procedure. The 3D groups are modeled in terms of grouped tensors and despekled with singular value decomposition. Moreover, a variant of the bilateral filter is used as a post-processing step to recover and enhance edges’ quality. Experimental results have demonstrated that the designed framework guarantees a good despeckling performance in ultrasound images according to the objective quality criteria commonly used in literature and via visual perception.

## 1. Introduction

The use of ultrasound images in diagnosing and assessing organs and soft tissue structure is well established. Because of its non-invasive, painless nature and computing improvements, ultrasound (US) is considered an essential medical imaging modality. The main issue disturbing US images is the existence of a random grainy pattern known as speckle, which is the primary factor that limits the performance in the diagnosis, detection, and classification problems. Speckle is conventionally described as a dominant source of noise in US imaging that decreases the contrast of soft tissue in the images and should be suppressed without reshaping any critical features of the images or altering relevant details. Since US imaging is a coherent sensing system, the speckle can be modeled as multiplicative noise that is also present in other types of images acquired by different sensors, such as lasers, synthetic aperture radar (SAR), and radiology, among others.

Over the years, numerous studies have been carried out to suppress speckle in US images. Long-established speckle reduction methods calculate the noise-free image through mathematical operations, such as the Lee filter [1] where the pixel’s values in the center of a spotted window are used along with its linear combination of average density. The filtered pixels are replaced with the new values obtained using the surrounding pixels. This method assures the despeckling while conserving the image’s sharpness and features. A similar classical filter is proposed by Kuan et al. [2] with the discrepancy of using a distinct signal model to calculate the values of the despeckled pixels, and the filtering is performed based on local statistics in the neighboring pixels. Another classical method is the Frost filter [3], which is based on the exponential function, whose parameters dwell on a local variation coefficient; this technique pursues a high-pass filter, mainly in high-contrast areas. Moreover, diverse approaches have been developed to define a threshold value for the coefficients of Lee and Frost filters [4].

In addition to these classical methods, several approaches based on the average, median, Gauss geometrical, Wiener, gamma maximum a posteriori (MAP), and Bayes–Gauss algorithms have been proposed for numerous applications [5,6]. The principal objective of these approaches is the suppression of speckle, conserving important features such as edges and fine details.

Besides traditional methods, new techniques have demonstrated a good performance in speckle suppression while preserving edges and details in medical US images. Garg and Khandelwal [7] presented a procedure for thresholding wavelet coefficients using the Neigh Shrink Sure filter and improving its performance via applying a bilateral filter. Rahimizadeh et al. [8] designed a weighting function in the neutrosophic domain for enhancing the performance of the non-local means (NLM) filter in reducing the speckle in US images. Here, the pixels that are characterized by three components, including truth membership, indeterminacy membership, and falsity membership, should be processed to measure the similarity between pixels. Sameera and Sudhish in [9] considered the statistical properties of US images by employing the time correlation between different video sequence frames to calculate the MAP estimation of a noisy pixel. In [10], Mei et al. introduced a despeckling method that utilized the non-local similarity using the optimized Bayesian NLM filter and a redundancy index of each pixel for determining which areas of an image have minimal redundancy. Zhang et al. [11] have developed a despeckling approach for high-frequency speckle components based on wavelet shrinkage for an additive speckle model that uses the statistical properties of the US image and a trilateral filter to suppress the speckle’s low-frequency components. Zhu et al. [12] introduced a despeckling method for US images that analyzed the local frequency information; this technique is invariant to the intensities of the amplitudes of the features. Wang et al. [13] devised a despeckling technique using stationary wavelet thresholding and applying an edge detector to the low-frequency sub-bands for preserving fine details.

An approach based on homomorphic filtering is given in Yaseen et al. [14] where total variation (TV) regularization is used over a dictionary trained by K -singular value decomposition (K-SVD) and optimized with the split Bregman algorithm. In [15], the DLRA filter is introduced for speckle reduction in US images, utilizing a low-rank approximation matrix and the weighted nuclear norm minimization based on the mathematical operation of third-order tensors. Nevertheless, this approach does not consider statistical information for creating the tensors. On the contrary, Nadeem et al. [16] differentiated between homogeneous areas and non-homogeneous ones by considering statistical properties. However, their results tended to modify the intensity of the pixels. Jubairhamed et al. [17] proposed a despeckling method in the anisotropic diffusion scheme. They utilized contourlet for thresholding the speckled pixels; nevertheless, the initial decomposition levels of the transformation approximate the original image, but the last decomposition level presents little relevance to the original image. In the study [18], a method for filtering images contaminated by additive-multiplicative noise is introduced using the formation of similar structures in 3D space, homomorphic transformation, where the 3D filtering approach is based on the sparse representation in the discrete cosine transform. This scheme employs a post-processing step that consists of a bilateral filter. More recently, Wang et al. [19] proposed a despeckling method for SAR images implementing a clustering approach based on the superpixels algorithm. They considered that the pixels have the same characteristics to furnish different weights for the speckle coefficients. In the work [20], a technique for despeckling SAR images has been introduced based on clustering areas using segmentation via k-means and MAP estimation.

Dabov et al. [21] devised a new framework for filtering Gaussian white noise in images. This approach is based on the block matching procedure, which is used for detecting movement in video sequences. First, a 3D group composed of similar patches in the images is formed by determining the Euclidean distance between them and a reference one. Then, the Wiener filter is used to obtain the denoised image. The BM3D framework has been used for different applications, such as despeckling SAR images [22,23,24]. Nevertheless, its performance decreases at higher noise levels and does not consider the different characteristics of the noise under the domain of the transformation employed, such as the discrete cosine transform (DCT). These drawbacks of the BM3D algorithm are addressed by Hasan and El-Sakka [25]. Furthermore, Santos et al. [26] provided a scheme for arranging the 3D groups by considering different stochastic distances (Kullback–Lebler distance, Rényi distance, Hellinger distance, and Bhattacharyya distance) for measuring the similarity between patches. They took into account the statistical properties of the US images; nonetheless, their method did not consider other features for comparing the patches.

It can be observed that different approaches take advantage of the fact that pixels’ intensities in local neighboring pixels of a clustered area are highly correlated. Consequently, this work aims to proportionate a despeckling method for medical ultrasound images that considers the images’ statistical properties in combination with their texture patterns for creating 3D groups in the BM3D scheme. For this purpose, the segmentation of US images is carried out considering superpixels and a variation of local binary patterns, named CMI-3DSVD (clustered-based using mutual information and 3D-singular value decomposition), permitting to improve the performance of the block matching procedure. Furthermore, the speckle reduction is modeled as a low-rank tensor approximation problem. The principal contributions of the proposed method are listed below.

1. The method integrates the local statistical properties and texture on a clustered area of an image based on superpixel segmentation and a variant of the local binary patterns, enhancing the performance of the block matching procedure in the BM3D framework.
2. The new approach uses the mutual information measure for comparing the similarity between patches in the block matching procedure to consider the high correlated information on a clustered area of a medical US image for the despeckling procedure.
3. The proposed arrangement from the 3D grouping in the block matching algorithm supports an efficient procedure for despeckling using tensor algebra.
4. A post-processing stage is introduced by employing a variant of the bilateral filter to increase the quality of edges and fine details in medical ultrasound images.

The rest of this paper is organized as follows. Section 2 explains the proposed despeckling method. Later, the experimental results are described and discussed in Section 3. Finally, this paper concludes in Section 4.

## 2. Material and Methods

Since the medical ultrasound images are acquired from a coherent acquisition system, the presence of speckle can be modeled as multiplicative noise. Such a model has been widely used for deploying speckle reduction methods and can be expressed as follows.

(1) X(i,j)=Y(i,j)S(i,j)+A(i,j),

where X(i,j) corresponds to the image corrupted with speckle, S(i,j) denotes the speckle coefficients in the image, and Y(i,j) is the noise-free image. A(i,j) represents additive noise, which usually is present in a slight amount. Finally, (i,j) represents the spatial position of the pixels.

**Figure 1:**
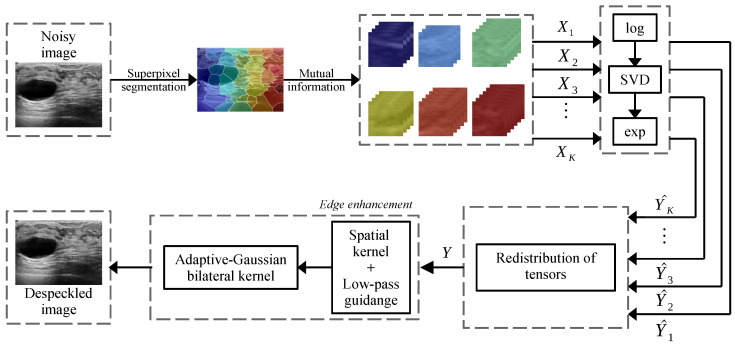
Block diagram of the proposed CMI-3DSVD filter.

The proposed method can be divided into three principal stages. Firstly, the image is segmented to improve the performance of the block matching procedure and the tensorization of the image. The second stage consists of the filtering operation of an image by using the minimization of the low-rank approximation. Finally, the enhancement of the edges and fine details are performed in the third stage. Figure 1 illustrates the proposed method via a block diagram.

### 2.1. Image Segmentation

**Figure 2:**
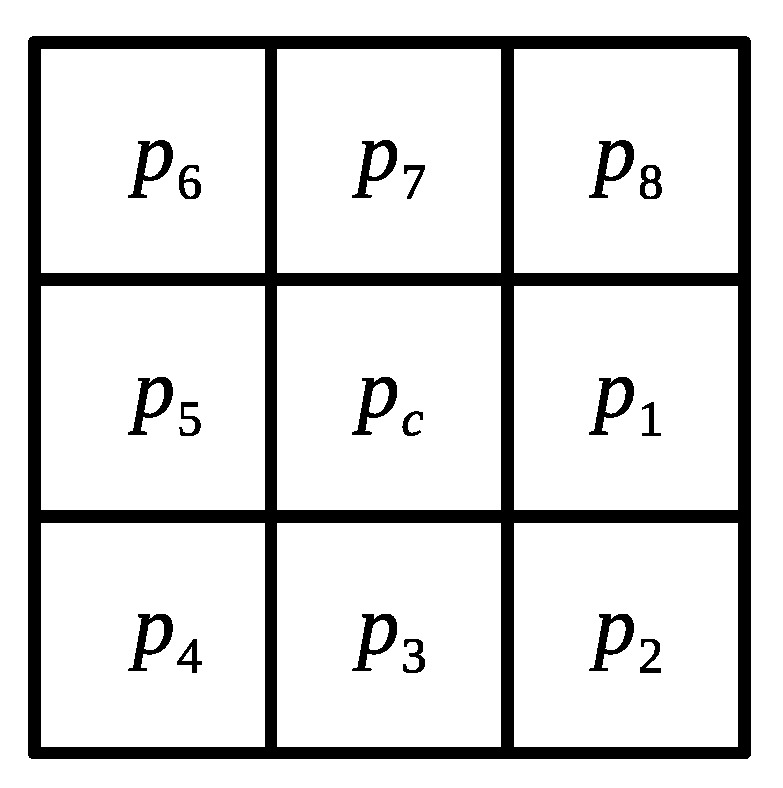
Patch of size 3×3 used for the extraction of texture.

In order to limit the search for similar patches and contemplate the local statistical properties and texture features, a segmentation scheme is proposed based on the superpixels algorithm. Therefore, the local binary pattern (LBP) algorithm is considered because it extracts irregular texture features supporting rotation invariance in grayscale, such as in medical images [27]. The local tri-directional pattern (LTriDP) [28] is an expansion of LBP that employs the relation between a center pixel and the neighboring pixels in a window of size of 3×3 pixels, as shown in Figure 2.

The magnitude of the LTriDP is calculated to determine a pixel’s texture feature, considering the difference of the grayscale value between the central pixel $p_{c}$ and the neighboring pixels $p_{t}$ in the vertical and horizontal directions. This process is described as follows:

(2) $$\begin{array}{c} M_{1}=\sqrt{{\left(p_{t-1}-p_{c}\right)}^{2}+{\left(p_{t+1}-p_{c}\right)}^{2}}\\ M_{2}=\sqrt{{\left(p_{t-1}-p_{c}\right)}^{2}+{\left(p_{t+1}-p_{t}\right)}^{2}}, \end{array}$$

where t=1,2,…,8; in the specific case when t=1 the pixel $p_{t-1}=p_{8}$. According to the values obtained from (2), it is possible to determine the magnitude of this pixel based on the results of $M_{1}$ and $M_{2}$ using the following equation:

(3) $$\mathrm{Mag}\left(t\right)=\left\{\begin{array}{cc} 1, & M_{1}\geq M_{2}\\ 0, & M_{1}<M_{2}. \end{array}\right.$$

The local texture feature of the central pixel $p_{c}$ is calculated as

(4) $${\mathrm{LTriDP}}_{{\mathrm{Mag}}_{\left(p_{c}\right)}}=\left\{\mathrm{Mag}\left(1\right),\mathrm{Mag}\left(2\right),\ldots ,\mathrm{Mag}\left(8\right)\right\},$$

(5) $$\mathrm{LTriDP}\left(p_{c}\right)=\sum_{t=0}^{7}2^{t}\times {\mathrm{LTriDP}}_{{\mathrm{Mag}}_{\left(p_{c}\right)}}.$$

Once the texture features are computed, the simple linear iterative clustering (SLIC) method is applied. It generates superpixels employing the color similarity and the proximity of the pixels of an image. It is based on the K-means clustering procedure to reduce the searching area [29]. However, Kim et al. [30] concluded that only the grayscale pixels that are similar to the center of the cluster are used to update the spatial position of the center pixel.

Algorithm 1 explains the procedure for the SLIC superpixel segmentation, where $d_{*}$ denotes a distance between the following features of the pixel $p_{t}$ and the central pixel $C_{K}$: *g* is the pixel’s grayscale value, *S* is spatial distance, and *T* defines the LTriDP value. Moreover, $N_{g}$ is the maximum distance of $d_{g}$, $N_{S}$ represents the maximum size of the neighborhood of the cluster ($N_{S}=S$), and $N_{T}$ is the maximum of $d_{T}$.
**Algorithm 1** Segmentation based on superpixels. **Input:** noisy image X(i,j) **Output:** segmented image $\bar{X}\left(i,j\right)$1:Find the number of peaks in the histogram to define the *K* clusters.Calculate the size of the cluster *S* by:2:$S=\sqrt{P/K}$;                    ▹*P* is the number of pixels.Determine the distance for clustering in a segment of size of 2S×2S:3:$d_{g}=\sqrt{{\left(g_{C_{K}}-g_{p_{t}}\right)}^{2}}$4:$d_{S}=\sqrt{{\left(i_{C_{K}}-i_{p_{t}}\right)}^{2}+{\left(j_{C_{K}}-j_{p_{t}}\right)}^{2}}$5:$d_{T}=\sqrt{{\left(T_{C_{K}}-T_{p_{t}}\right)}^{2}},$           ▹*T* is the magnitude ${\mathrm{LTriDP}}_{{\mathrm{Mag}}_{\left(p_{c}\right)}}.$6:$D=\sqrt{{\left(\frac{d_{g}}{N_{g}}\right)}^{2}+{\left(\frac{d_{S}}{N_{S}}\right)}^{2}+{\left(\frac{d_{T}}{N_{T}}\right)}^{2}}$Update the clusters by:7:$G_{K}=\frac{1}{P_{K}}\sum_{r\in \alpha_{K}}G_{r}$8:$S_{K}=\frac{1}{P_{K}}\sum_{r\in \alpha_{K}}S_{r}$9:$\bar{X}\left(i,j\right)$ is composed by clusters $X_{0}$ to $X_{K}$

In the previous pseudocode, $P_{K}$ defines the total number of pixels in the *K*-th superpixel $\alpha_{K}=\left(\right|C_{K}-p_{t}\left|<\sigma_{K}\right)\cap O_{K}$; here, $O_{K}$ is the set of clustered pixels, and $\sigma_{K}$ denotes its standard deviation.

### 2.2. Block Matching via Mutual Information

The next stage in the proposed method consists of searching for similar patches to the reference one via block matching utilizing the mutual information (MI) measure, which characterizes the statistical dependence between two random variables. The MI measure, denoted as $\mathrm{MI}(X_{0},X_{k})$, is the average amount of information provided by the occurrence of the reference patch $X_{0}$ on the occurrence of the *k*-th similar patch $X_{k}$ of a fixed size in a clustered area. Contemplating that a probability density function represents the speckle coefficients intensity and pixel values, it is viable to quantify the similarity between patches through the entropy of this distribution [31]. The MI measure is expressed as follows:

(6) $$\mathrm{MI}\left(X_{0},X_{k}\right)=H\left(X_{0}\right)+H\left(X_{k}\right)-H\left(X_{0},X_{k}\right),$$

where $H(X_{0})$ and $H(X_{k})$ represent the entropy of $X_{0}$ and $X_{k}$, respectively; $H(X_{0},X_{k})$ is the joint entropy between them. $H(X_{0})$ is defined as follows:

(7) $$H\left(X_{0}\right)=-\sum_{t}P\left(p_{t}\right)\log_{2}P\left(p_{t}\right),$$

where $P(p_{t})$ is the probability of the *t*-th pixel *p*. Then, the joint entropy is defined as follows:

(8) $$H\left(X_{0},X_{k}\right)=-\sum_{t}P\left(X_{0}=X_{0_{t}},X_{k}=Y_{k_{t}}\right)\log_{2}P\left(X_{0}=X_{0_{t}},X_{k}=X_{k_{t}}\right).$$

Based on the block matching algorithm, a search for similar patches maximizing the mutual information is performed. The patches are composed of the pixels with the maximum dependence between them since the mutual information depends on the pixel’s probability of occurrence between the blocks.

The entropy $H(X_{0})$ and $H(X_{k})$ can be found by computing the histograms that arrange the total number of pixels analyzed for every possible intensity found in a patch. Then, this histogram is normalized by the total number of pixels to estimate the probability density function.

The joint two-dimensional histogram between two blocks is calculated to find the joint entropy $H(X_{0},X_{k})$. The joint histogram is akin to the one-dimensional histogram. However, the first dimension corresponds to the intensities of a reference patch, while the second one represents the intensities of the *k*-th reference patch.

As a result, we obtain a third order tensor, denoted as $X_{K}\left(i,j,k\right)$, where the subscript *K* defines the *K*-th clustered area of the input image.

### 2.3. Despeckling via Singular Value Decomposition

One of the most concurrent approaches for despeckling images is under the homomorphic transform domain, which is based on calculating the logarithm in order to remodel the multiplicative nature of the speckle into an additive model, such as:

(9) $$\begin{array}{c} \log\left[X_{K}\left(i,j\right)\right]=\log\left[Y_{K}\left(i,j\right)S_{K}\left(i,j\right)+A_{K}\left(i,j\right)\right],\\ {\dot{X}}_{K}\left(i,j\right)={\dot{Y}}_{K}\left(i,j\right)+{\dot{S}}_{K}\left(i,j\right)+\frac{A_{K}\left(i,j\right)}{Y_{K}\left(i,j\right)S_{K}\left(i,j\right)}, \end{array}$$

where ${\dot{S}}_{K}\left(i,j\right)$ represents the speckle in an additive log-transformed model, which is usually considered for homomorphic schemes, ${\dot{Y}}_{K}\left(i,j\right)$ denotes a noise-free 3D block and ${\dot{X}}_{K}\left(i,j\right)$ corresponds to a speckled 3D block, and the last term reflects the influence of additive noise with sufficiently low intensity compared to speckle coefficients $\left(\frac{A_{K}\left(i,j\right)}{Y_{K}\left(i,j\right)S_{K}\left(i,j\right)}\ll 1\right)$. Here, the subscript *K* represents the tensor that corresponds to the *K*-th clustered area of the input image, as shown in Figure 1.

The weighted nuclear norm minimization (WNNM) is a low-rank approximation technique utilized to estimate the despeckled image from grouped blocks. The WNNM framework is defined as

(10) $${\hat{\dot{Y}}}_{K}=\arg\min_{{\dot{Y}}_{K}}\frac{1}{{\dot{\sigma }}_{n}^{2}}{\left|\left|{\dot{X}}_{K}-{\dot{Y}}_{K}\right|\right|}_{F}^{2}+{\left|\left|{\dot{Y}}_{K}\right|\right|}_{w,*},$$

where ${\dot{\sigma }}_{n}^{2}$ indicates the noise variance under the additive model, and ${\left|\left|{\dot{X}}_{K}-{\dot{Y}}_{K}\right|\right|}_{F}^{2}$ is the *F*-norm. The solution to the previous equation is given by

(11) $${\hat{\dot{Y}}}_{K}={\mathrm{US}}_{w}\left(\Sigma \right)V^{T}.$$

The proof has been explained and discussed in [32] that corresponds to the singular value decomposition of the noised patch ${\dot{X}}_{K}$, such as $\mathrm{SVD}\left({\dot{X}}_{K}\right)=U\Sigma V^{T}$. Here, Σ corresponds to the singular values of ${\dot{X}}_{K}$. Further, the operation of soft-thresholding $S_{w}\left(\Sigma \right)$ is obtained as follows:

(12) $$S_{w}\left(\Sigma_{K}\right)=\max\left(\left(\Sigma_{K}-w_{K}\right),0\right).$$

Here, $w_{K}$ defines the weights of the singular values and are formulated as

(13) $$w_{K}=\frac{r\sqrt{k}}{\left(\sigma_{K}\left(X_{K}\right)+\epsilon \right)},$$

where r>0, *k* defines the number of similar patches in the third order tensor, $\sigma_{K}$ is the noise variance in the log-domain, and $\epsilon =10^{-16}$ is a small constant to avoid the division by zero. Afterwards, every despeckled tensor ${\hat{\dot{Y}}}_{K}$ is subjected to the exponential transformation to return to the original model of the image, resulting in

(14) $${\hat{Y}}_{K}=\exp\left({\hat{\dot{Y}}}_{K}\right).$$

Finally, for the destensorization of the despeckled patches, the values obtained from the MI are used as weight values according to the following equation:

(15) $$\hat{Y}\left(i,j\right)=\frac{\sum_{m=1}^{k}{\hat{Y}}_{K}\left(i,j,k\right)q_{m}}{\sum_{m=1}^{k}q_{m}},$$

where ${\hat{Y}}_{K}\left(i,j,k\right)$ corresponds to the *K*-th despeckled tensor ${\hat{Y}}_{K}$ formed by *k*-th similar patches; and $q_{m}=1-\mathrm{MI}\left(X_{K}\left(i,j,0\right),X_{K}\left(i,j,m\right)\right)$. Algorithm 2 describes the procedure for despeckling a tensor using the SVD.
**Algorithm 2** Despeckling tensors via SVD.     **Input:** noisy tensor $X_{K}$     **Output:** despeckled tensor ${\hat{Y}}_{K}$1:${\dot{X}}_{K}=\log\left(\hat{X}\right)$                ▹ log transform2:**for**K=1 to *K*-th cluster **do**3:     Estimate weight vector *w*4:     $w_{K}=\frac{r\sqrt{p}}{\sigma_{K}\left(X_{K}\right)+\epsilon }$5:     Singular value decomposition: $\left|U,\Sigma ,V|=\mathrm{SVD}({\dot{X}}_{K}\right)$;6:     Get the estimation: ${\dot{Y}}_{K}=US_{w}\left(\Sigma \right)V^{T}.$;7:     $S_{w}\left(\Sigma_{K}\right)=\max\left(\left(\Sigma_{K}-w_{K}\right),0\right).$8:     ${\hat{Y}}_{K}=\exp\left({\dot{Y}}_{K}\right)$              ▹ exp transform9:**end for****Return:** ${\hat{Y}}_{K}$

### 2.4. Edge and Fine Detail Enhancement

In the final stage, named the post-processing phase, the Gaussian-Adaptive Bilateral Filter (GABF) [33] is used to preserve and improve the quality of the edges and fine details. The principal idea of this filter consists of producing low-pass Gaussian guidance. A weighted average of the pixels achieves this in the adjacent position, with a weight descending from the center.

### 2.5. Algorithm Summary

The proposed filtering technique consists of three principal stages: (i) segmentation based on superpixels and block matching via mutual information, (ii) despeckling of 3D blocks via singular value decomposition, and (iii) enhancement of edges and fine details. A detailed description of the proposed CMI-3DSVD filter is summarized in the Algorithm 3, where these operations are combined in the despeckling of US images.

### 2.6. Image Quality Metrics

To evaluate the performance of the proposed despeckling method, the following convention to describe the objective criteria is used: $X_{d}$ is the despeckled image, $X_{n}$ denotes the noisy image, and $X_{g}$ represents the ground truth noiseless image; *M* and *N* are the dimensions of the image. Finally, the denotations E[·] and VAR[·] indicate the expected value and variance operations. The criteria that require $X_{g}$ are known as full-reference metrics, while the criteria that only use $X_{d}$ and $X_{n}$ are the non-reference metrics [26].
**Algorithm 3** Algorithm summary: CMI-3DSVD filter.**(i) Segmentation based on superpixels**       **Input:** noisy image X(i,j)       **Output:** segmented image $\bar{X}\left(i,j\right)$1:Algorithm 1**Block matching via mutual information:**       **Input:** segmented image $\bar{X}\left(i,j\right)$       **Output:** noisy tensor $X_{K}\left(i,j,k\right)$       Searching of the *k*-th similar patches to the reference one $X_{0}$ via:1:**for**K=1 to *K*-th cluster **do**2:    **for** k=1 to *k*-th similar patch **do**3:     $\mathrm{MI}\left(X_{0},X_{k}\right)=H\left(X_{0}\right)+H\left(X_{k}\right)-H\left(X_{0},X_{k}\right)$4:        **if** $\mathrm{MI}(X_{0},X_{K})<\mathrm{threshold}$5:        $X_{K}\left(i,j,k\right)=\left[X_{0};\ldots ;X_{k}\right]$            ▹ stacking of similar patches6:    **end for**7:**end for****(ii) Despeckling of tensors via Singular Value Decomposition**       **Input:** noisy tensor $X_{K}\left(i,j,k\right)$       **Output:** despeckled tensor ${\hat{Y}}_{K}\left(i,j,k\right)$1:Algorithm 2**Redistribution of tensors (destensorization):**       **Input:** despeckled tensor ${\hat{Y}}_{K}\left(i,j,k\right)$       **Output:** despeckled image Y(i,j)       Searching of the *k*-th similar patches to the reference one $X_{0}$ via:1:**for**K=1 to *K*-th cluster **do**2:    **for** k=1 to *k*-th similar patch **do**3:        $\hat{Y}\left(i,j\right)=\frac{\sum_{m=1}^{k}{\hat{Y}}_{K}\left(i,j,k\right)q_{m}}{\sum_{m=1}^{k}q_{m}}$4:    **end for**5:**end for****(iii) Enhancement of edges and fine details**       **Input:** despeckled image ${\hat{Y}}_{K}\left(i,j\right)$       **Output:** enhanced and despeckled image Y(i,j)       Gaussian-adaptive bilateral filter:1:Estimate the spatial kernel2:Low-pass guidance3:$Y\left(i,j\right)=\mathrm{GABF}(\hat{Y}\left(i,j\right))$
*(A) Speckle smoothing metrics*. Within homogeneous regions of an image, the speckle intensity, also known as the speckle index (SI), is given by the relation of the standard deviation and the mean [34] as follows:

(16) $$\mathrm{SI}=\frac{\sqrt{\mathrm{VAR}[X_{n}]}}{E[X_{n}]}.$$

The speckle suppression index (SSI) is obtained by normalizing the SI of the despeckled image by the SI of the original image in a specific homogeneous area in an image, which is formulated by

(17) $$\mathrm{SSI}=\left(\frac{\sqrt{\mathrm{VAR}[X_{d}]}}{E[X_{d}]}\right)\left(\frac{E[X_{n}]}{\sqrt{\mathrm{VAR}[X_{n}]}}\right)$$

The lower the SSI is, the better despeckling performance the filter has. Thus, SSI<1. Nonetheless, the SSI may fail to evaluate the speckle removal performance if the filter overestimates the despeckled image mean. To avoid this issue, the speckle suppression and mean preservation index (SMPI) are used:

(18) $$\mathrm{SMPI}=\left(R+E\left[X_{n}\right]-E\left[X_{d}\right]\right)\left(\frac{\sqrt{\mathrm{VAR}[X_{d}]}}{\sqrt{\mathrm{VAR}[X_{n}]}}\right),$$

where

$$R=\frac{\max\left(E[X_{d}]\right)-\min\left(E[X_{d}]\right)}{E[X_{n}]}.$$

A lower value of SMPI indicates a better despeckling performance regarding mean preservation and speckle reduction [35].

*(B) Peak signal-to-noise ratio*. The peak signal-to-noise ratio (PSNR) [36] is the ratio between the power of a signal and the power of the disturbing noise and is expressed as

(19) $$\mathrm{PSNR}=10\log_{10}\left(\frac{255^{2}}{\mathrm{MSE}}\right).$$

The mean square error (MSE) represents the noise power and may be calculated as

(20) $$\mathrm{MSE}=\frac{1}{MN}\sum_{i,j=1}^{M,N}{\left(X_{g}\left(i,j\right)-X_{d}\left(i,j\right)\right)}^{2}.$$

A high PSNR establishes a high signal-to-noise ratio and, consequently, a better filtering performance.

*(C) Structural similarity index*. The structural similarity index (SSIM) is a quality measure index that compares two images in terms of structures, luminance, and contrast. In order to implement the SSIM as a full-reference criterion, Taxt [37] suggests using the ground truth noiseless image. The SSIM values range in the interval [0,1], where 0 indicates total dissimilarity and 1 total similarity.

*(D) Edge preservation index*. The edge preservation index (EPI) is a criterion that specifies the effectiveness of preserving edges of a filtering method. In this work, there is used the definition given by Sattar et al. [38]

(21) $$\mathrm{EPI}=\frac{\sum_{i,j=1}^{M,N}\left(H_{g}\left(i,j\right)H_{d}\left(i,j\right)\right)}{\sqrt{\left(\sum_{i,j=1}^{M,N}H_{g}{\left(i,j\right)}^{2}\right)\left(\sum_{i,j=1}^{M,N}H_{d}{\left(i,j\right)}^{2}\right)}},$$

where $H_{g}=\left(\Delta X_{g}-E\left[\Delta X_{g}\right]\right)$, $H_{d}=\left(\Delta X_{d}-E\left[\Delta X_{d}\right]\right)$ and Δ corresponds to the high-pass filtered image, which is obtained with a 3×3 pixel standard approximation of the Laplacian operator. The EPI values extend between [0,1], where the values near 1 indicate a good conservation of edges.

*(E) Resolution α*. It is a metric of the resolution of US images, which has been employed such as Santos et al. [26]. It is computed as the percentage of pixels in the auto-correlation function of the despeckled image that outstrips 75% of its maximum value. A lower resolution value (α) indicates a better image resolution.

## 3. Experimental Results and Discussion

**Figure 3:**
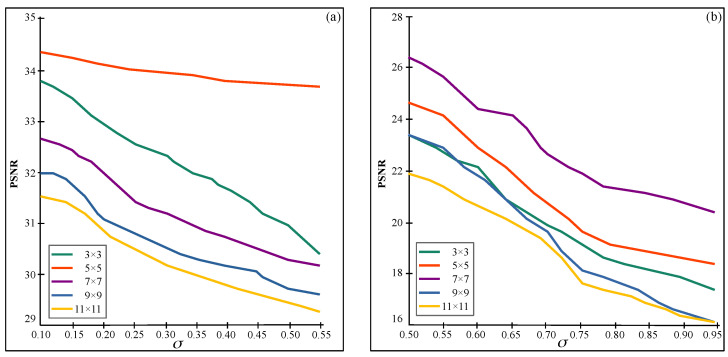
PSNR values that validate the optimum size of patches for the tensorization of the image. (**a**) For σ≤0.50 and (**b**) for σ>0.50.

During the development of the proposed method, numerous experiments were carried out to obtain an optimal window size for 3D filtering considering the properties and the PSNR of the filtered image. According to the tests carried out by Dabov et al. [21], two profiles were considered according to the intensity of the speckle coefficients, since at high noise levels, the images are deformed on a larger scale. The noise was tested for a standard deviation of different levels of multiplicative speckle noise ranging from 0.10 to 1.0. The results represented in Figure 3 indicates that the optimum size for σ≤0.50 is size of 5×5, while 7×7 is for σ>0.50.

**Figure 4:**
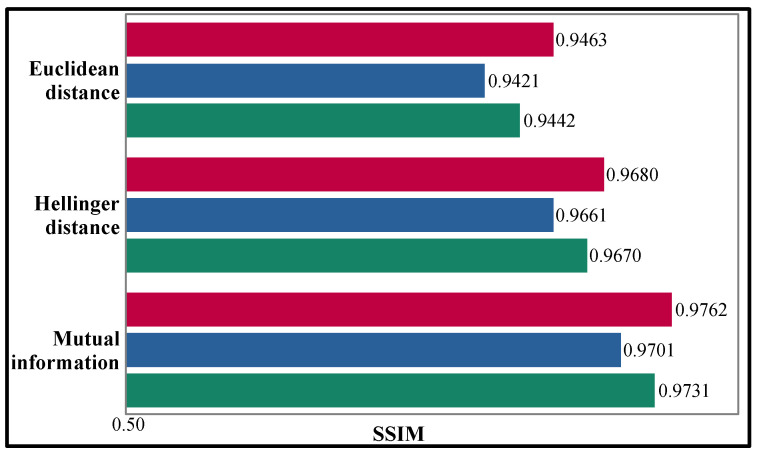
Evaluation of the block matching performance considering the SSIM of Euclidean distance, Hellinger distance, and mutual information. Minimum number of similar patches (red) vs. maximum number of similar patches (blue) and its mean value (green).

To give a view of the performance of the block matching procedure with the minimum and the maximum number of stacked elements using the Euclidean and Hellinger distances and the proposed mutual information, the SSIM values between the reference patch and its similar patches are illustrated in Figure 4. Although the difference in SSIM values is minimal, it is possible to determine that the mutual information yields better performance for constituting third-order tensors.

The proposed approach has been validated using simulated and authentic ultrasound images. For all cases, we compare our approach with the BM3D [21] method, SD-BM3D method (Hellinger distance) [26], DLRA method [9], K-SVD [14] method, and Contourlet-based method (CLT) [17], which have been described in Section 1. However, each filter has been stated with different parameters in the reports mentioned above, managing the compensation between smoothing and detail preservation; thus, a rule can not be set to determine the optimum set of parameters to be used in general. Consequently, the parameters for experimentation with the filters have been chosen to overcome this issue by adopting the following procedure. First, the BM3D filter is run with a set of parameters that visually seems to have a good balance between filtering and edge preservation. Second, calculate the resolution (α) metric and implement the other filters, modifying the parameters until the same resolution (α) is known. Under these considerations, the performance of the filters is optimum to be compared, contemplating that the images have the same resolution metric. The experimental procedure was executed in an Intel Core i7-6700K PC with Windows 10, using the MATLAB 2016b environment.

### 3.1. Experiments with Simulated Ultrasound Images

**Figure 5:**
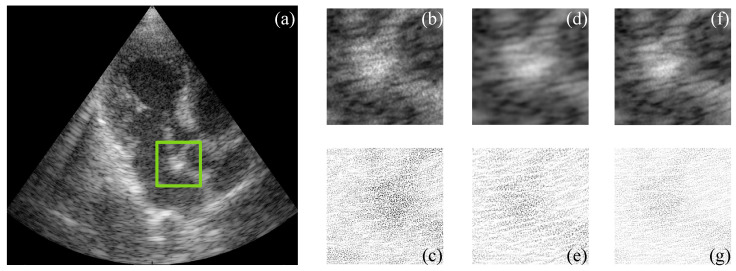
Subjective visual results of the despeckled simulated US image US-sim-04. (**a**) Input image. Details are taken from the region marked in green: BM3D: (**b**) despeckled image, PSNR = 15.47 dB, EPI = 0.2791; (**c**) error image. SD-BM3D: (**d**) despeckled image, PSNR = 16.07 dB, EPI = 0.2898; (**e**) error image. CMI-3DSVD: (**f**) despeckled image, PSNR = 16.35 dB, EPI = 0.3156; (**g**) error image.

**Figure 6:**
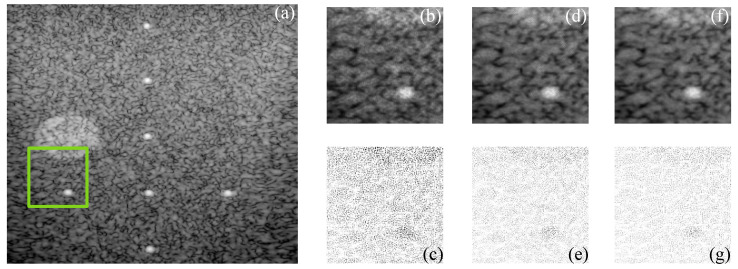
Subjective visual results of the despeckled simulated US image US-sim-11. (**a**) Input image. Details are taken from the region marked in green: BM3D: (**b**) despeckled image, PSNR = 17.02 dB, EPI = 0.3247; (**c**) error image. SD-BM3D: (**d**) despeckled image, PSNR = 17.37 dB, EPI = 0.3516; (**e**) error image. CMI-3DSVD: (**f**) despeckled image, PSNR = 18.04 dB, EPI = 0.4021; (**g**) error image.

For the simulated US images, we used the tool Field II [39,40] to create images for the cyst phantom, such as indicated in [26]. There are three cyst regions, which are the black areas of the Figure 5, their amplitude is distributed to the corresponding unit mean Gaussian distribution. There are also high scattering regions, represented by white spots in Figure 6 whose amplitudes are multiplied by ten and are made zero inside the cyst region. The configuration to simulate ultrasound images using the Field-II program is given in Table 1.

**Figure 7:**
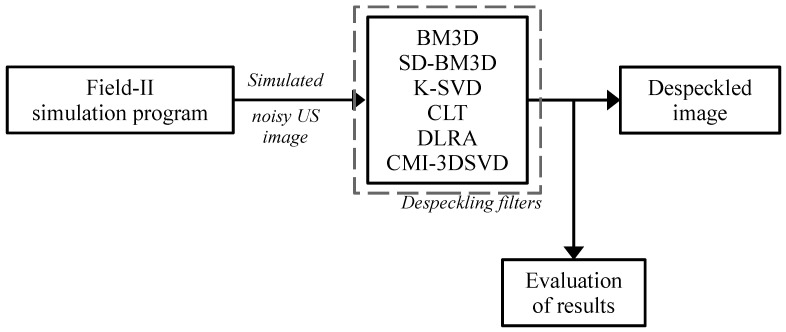
Block diagram of the filtering testing procedure for simulated US images.

The density of 10 scatterers/mm^3^ is used to achieve fully developed speckle, which is confirmed to be constant by checking the statistics of the speckle that should correspond to a Rayleigh distribution in a homogeneous region. According to the experiments, and for the Field-II configuration, as shown in Table 1, a density higher than 8 scatterers/mm^3^ would be enough to generate a fully developed speckled ultrasound image. The simulated speckled US images are filtered via the proposed CMI-3DSVD despeckling framework, according to the algorithm summary (Section 2.5), and compared with the state-of-the-art approaches. Figure 7 illustrates the process performed for testing, evaluating, and comparing the results of the despeckled simulated US images using the comparing methods and the CMI-3DSVD filter. This procedure is performed ten times to average the objective criteria values.

Figure 5 and Figure 6 show the simulated and filtered ultrasound images, indicated as US-sim-04 and US-sim-11, respectively, with the subjective visual comparison with the BM3D and SD-BM3D.

The visual results in Figure 5 and Figure 6 show that the designed framework can achieve good performance for the despeckling procedure. As can be observed, the BM3D technique produces artifacts that degrade the processed images, and the SD-BM3D method tends to over-smooth edges and fine details by comparing the error images. The principal disadvantage of these techniques is that they do not consider the images’ local statistical properties and texture features for grouping the 3D blocks, which limits the denoising performance. The proposed CMI-3DSVD filter outperforms the mentioned denoising methods by preserving important details of the image, such as edges, and by not blurring homogeneous regions.

Table 2 presents the objective results for the criteria values of PSNR, SSIM, and EPI, and Table 3 exposes the values of SSI, SMPI, and resolution α for the complete database of simulated ultrasound images. The experiments were run ten times, and the criteria values were averaged.

Table 2 shows that the proposed method achieves the best results in the full-referenced metrics, indicating that the CMI-3DSVD filter carries out a despeckled image close to a noise-free one. It can be observed that the designed framework surpasses, on average, by 0.53 dB for PSNR, 0.013 for SSIM, and 0.020 for EPI the results of the state-of-the-art methods at different noise intensities. Table 3 exposes the non-referenced criteria values, demonstrating that the CMI-3DSVD despeckling framework suppresses the speckle coefficients without deforming the processed image, considering the highest values of SMPI. As shown in the previous tables, the proposed framework is robust to different noise intensities, demonstrating that it performs well for applications in detection systems and classifying pathologies through ultrasound images.

### 3.2. Experiments with Real Ultrasound Images

The experiments with real images were performed using a database of ultrasound frames of breast lesions recorded from malignant and benign tumors. This dataset is available in [41]. The complete database consists of 183 frames from malignant tumors and 183 from benign tumors, but ten images from each set of frames have been taken to evaluate the proposed method.

**Figure 8:**
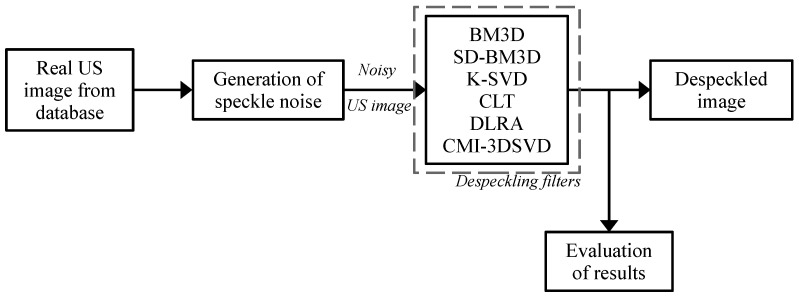
Experimental procedure for the comparison of the CMI-3DSVD filter on real ultrasound images.

The speckle noise was simulated employing the following procedure. First, two random vectors were created to generate the speckle coefficients presented in the real US image from the database. Afterward, two random vectors were formed with sizes corresponding to the width and height of the processed image to create a pure noisy image. The standard deviation of the noise varied between 0 and 1 in intervals of 0.1. Then, the pure noisy image is combined with the real US one considering the multiplicative model described in Equation (1). Thus, the noisy image is filtered using the proposed CMI-3SVD based on the algorithm summary (Section 2.5). Figure 8 illustrates the implementation process for testing and evaluating the CMI-3DSVD filter and comparing techniques. This procedure is performed ten times to average the objective criteria values.

**Figure 9:**
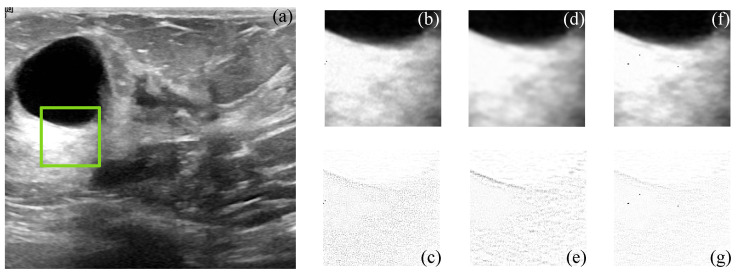
Subjective visual results of the despeckled real US image US-07. (**a**) Input image. Details are taken from the region marked in green: BM3D: (**b**) despeckled image, PSNR = 26.57 dB, EPI = 0.7164; (**c**) error image. DLRA: (**d**) despeckled image, PSNR = 26.92 dB, EPI = 0.8275; (**e**) error image. CMI-3DSVD: (**f**) despeckled image, PSNR = 27.10 dB, EPI = 0.8641; (**g**) error image.

**Figure 10:**
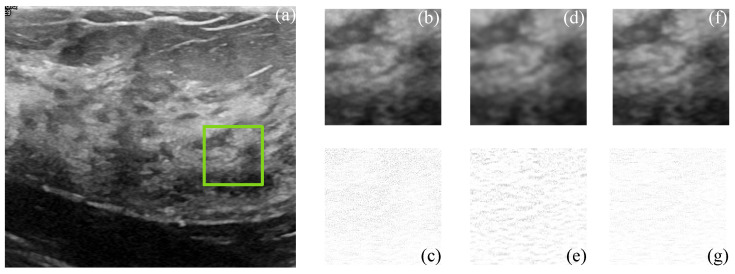
Subjective visual results of the despeckled real US image US-20. (**a**) Input image. Details are taken from the region marked in green: BM3D: (**b**) despeckled image, PSNR = 25.48 dB, EPI = 0.6872; (**c**) error image. SD-BM3D: (**d**) despeckled image, PSNR = 26.01 dB, EPI = 0.7158; (**e**) error image. CMI-3DSVD: (**f**) despeckled image, PSNR = 26.33 dB, EPI = 0.7396; (**g**) error image.

Figure 9 and Figure 10 show the actual filtered ultrasound images, named US-07 and US-20, respectively, with the subjective visual comparison of the proposed CMI-3DSVD against the BM3D and DLRA methods.

Figure 9 and Figure 10 show that the proposed approach provides high-quality despeckled images compared to the BM3D and DLRA methods. Moreover, as noticed in the error images, the DLRA filter causes blurring in detailed areas. This drawback resides in the lack of consideration of statistical properties for 3D group creation and the use of the Euclidean distance to measure the similarity between patches, which may cause alteration of the edges’ properties of the processed 3D block. In opposition, the CMI-3DSVD filter achieves better performance since it preserves edge quality and does not deform homogeneous regions in the processed images.

In order to validate the performance on speckled real US images of the CMI-3DSVD filter and compare it with other despeckling methods, Table 4 shows the objective criteria values of PSNR, SSIM, and EPI as Table 5, the values of SSI, SMPI, and resolution α.

Table 4 shows that the CMI-3DSVD filter produces the despeckled images with the best values of the full-referenced metrics, which means that it provides an image similar to a noise-free one. Thus, the proposed CMI-3DSVD filter outperforms on average by 0.60 dB for PSNR, 0.018 for SSIM, and 0.021 for EPI compared with state-of-the-art methods. Table 5 shows the non-referenced criteria values, which demonstrate that the designed framework reduces the intensity of the speckle coefficients while conserving the information of the processed image, considering the highest values of SSI and SMPI.

In addition to evaluating and comparing the filters using objective quality metrics, a subjective evaluation based on the experience of a medical radiologist is performed. For this, the same 20 real ultrasound images processed by the CMI-3DSVD and other comparison methods were used. Contemplating the subjective visual quality of the image filtered by each method, the specialist assigned them a score according to the following scale: 1—bad, 2—poor, 3—fair, 4—good, and 5—excellent without knowing which image corresponds to each filter. This scale is provided by the International Telecommunications Union (UTI) quality grading recommendations in [42]. The results of this evaluation are represented in Table 6 and demonstrate that the proposed CMI-3DSVD despeckling method achieves high-quality despeckled images, considering the subjective visual perception of a specialist.

## 4. Conclusions

This paper presents a novel despeckling method for medical ultrasound images. The consideration of the local statistical properties and texture on a clustered area of the images appears to demonstrate better performance with the block matching procedure, which implies an increased suppression of the speckle coefficients in homogeneous regions by comparing the results of the SD-BM3D and DLRA filters. Moreover, the proposed CMI-3DSVD achieves a good execution in preserving the edges and fine details, which are important features for the tasks of classification or detection.

Furthermore, using the mutual information as a similarity measure for the block matching procedure improves the formation of third-order tensors, enhancing the despeckling procedure via SVD. Moreover, the suggested post-processing stage enhances the edges and produces high-quality despeckled images, conserving relevant elements for the detection of tumors or the diagnosis of diseases through medical ultrasound images. Finally, a preliminary test employing the subjective visual perception of a radiology specialist was performed to evaluate the suitability of the proposed CMI-3DSVD despeckling method for applications in medicine, providing a higher score than the compared methods and achieving a category of high quality.

## Figures and Tables

**Table 1:** Field-II transducer configuration.

Transducer center frequency	$3.5\times 10^{6}$ Hz
Sampling frequency	$100\times 10^{6}$ Hz
Speed of sound	1540 m/s
Width of element	1540/($3.5\times 10^{6}$) m
Height of element	5/1000 m
Number of physical elements	192
Number of active elements	62

**Table 2:** Average values of PSNR, SSIM, and EPI for the database of simulated US images, where the highest values are highlighted in bold.

Metric	Method	Noise Level				
		0.2	0.4	0.6	0.8	1.0
PSNR	BM3D	22.76	20.81	20.11	19.52	18.83
	SD-BM3D	23.90	21.85	21.11	20.50	19.77
	K-SVD	22.93	20.96	20.25	19.66	18.97
	CLT	22.99	21.01	20.30	19.72	19.02
	DLRA	24.12	22.04	21.30	20.69	19.95
	CMI-3DSVD	**24.71**	**22.59**	**21.82**	**21.20**	**20.44**
SSIM	BM3D	0.5806	0.5412	0.5090	0.4916	0.4718
	SD-BM3D	0.6096	0.5681	0.5344	0.5161	0.4953
	K-SVD	0.5848	0.5450	0.5126	0.4951	0.4752
	CLT	0.5863	0.5464	0.5140	0.4964	0.4765
	DLRA	0.6150	0.5731	0.5391	0.5207	0.4997
	CMI-3DSVD	**0.6302**	**0.5873**	**0.5524**	**0.5335**	**0.5121**
EPI	BM3D	0.6645	0.6472	0.6269	0.6154	0.6086
	SD-BM3D	0.6976	0.6795	0.6581	0.6461	0.6389
	K-SVD	0.6693	0.6519	0.6314	0.6198	0.6130
	CLT	0.6711	0.6536	0.6331	0.6214	0.6147
	DLRA	0.7038	0.6855	0.6640	0.6518	0.6447
	CMI-3DSVD	**0.7212**	**0.7024**	**0.6803**	**0.6679**	**0.6605**

**Table 3:** Average values of SSI, SMPI, and resolution α for the database of simulated US images, where the highest values are highlighted in bold.

Metric	Method	Noise Level				
		0.2	0.4	0.6	0.8	1.0
SSI	BM3D	0.1008	0.1073	0.1122	0.1182	0.1268
	SD-BM3D	0.1031	0.1097	0.1146	0.1208	0.1296
	K-SVD	0.1018	0.1083	0.1133	0.1193	0.1280
	CLT	0.1029	0.1095	0.1145	0.1206	0.1294
	DLRA	0.1074	0.1144	0.1195	0.1259	0.1351
	CMI-3DSVD	**0.1134**	**0.1208**	**0.1262**	**0.1329**	**0.1427**
SMPI	BM3D	0.2090	0.2044	0.1987	0.1969	0.1926
	SD-BM3D	0.2136	0.2089	0.2031	0.2013	0.1969
	K-SVD	0.2110	0.2063	0.2005	0.1983	0.1945
	CLT	0.2134	0.2086	0.2028	0.2010	0.1967
	DLRA	0.2228	0.2178	0.2117	0.2099	0.2053
	CMI-3DSVD	**0.2354**	**0.2299**	**0.2235**	**0.2216**	**0.2167**
α	BM3D	0.1408	0.1397	0.1370	0.1348	0.1337
	SD-BM3D	0.1468	0.1451	0.1427	0.1405	0.1382
	K-SVD	0.1422	0.1410	0.1383	0.1360	0.1349
	CLT	0.1437	0.1426	0.1398	0.1375	0.1364
	DLRA	0.1501	0.1489	0.1460	0.1437	0.1424
	CMI-3DSVD	**0.1584**	**0.1572**	**0.1541**	**0.1517**	**0.1504**

**Table 4:** Average values of PSNR, SSIM, and EPI for the database of real US images, where the highest values are highlighted in bold.

Metric	Method	Noise Level				
		0.2	0.4	0.6	0.8	1.0
PSNR	BM3D	28.99	26.51	25.61	24.87	23.99
	SD-BM3D	30.44	27.83	26.89	26.11	25.19
	K-SVD	29.21	26.70	25.79	25.05	24.16
	CLT	29.29	26.77	25.86	25.12	24.23
	DLRA	30.72	28.08	27.13	26.35	25.42
	CMI-3DSVD	**31.47**	**28.77**	**27.80**	**27.00**	**26.04**
SSIM	BM3D	0.7395	0.6893	0.6483	0.6261	0.6009
	SD-BM3D	0.7764	0.7236	0.6806	0.6573	0.6309
	K-SVD	0.7448	0.6941	0.6529	0.6306	0.6053
	CLT	0.7468	0.6960	0.6547	0.6323	0.6069
	DLRA	0.7833	0.7300	0.6867	0.6632	0.6365
	CMI-3DSVD	**0.8026**	**0.7480**	**0.7036**	**0.6795**	**0.6522**
EPI	BM3D	0.8463	0.8243	0.7984	0.7838	0.7752
	SD-BM3D	0.8885	0.8654	0.8382	0.8229	0.8138
	K-SVD	0.8524	0.8303	0.8042	0.7894	0.7808
	CLT	0.8547	0.8325	0.8063	0.7915	0.7829
	DLRA	0.8964	0.8731	0.8457	0.8302	0.8211
	CMI-3DSVD	**0.9185**	**0.8946**	**0.8665**	**0.8507**	**0.8413**

**Table 5:** Average values of SSI, SMPI, and resolution α for the database of real US images, where the highest values are highlighted in bold.

Metric	Method	Noise Level				
		0.2	0.4	0.6	0.8	1.0
SSI	BM3D	0.1401	0.1491	0.1559	0.1642	0.1762
	SD-BM3D	0.1432	0.1524	0.1593	0.1678	0.1800
	K-SVD	0.1414	0.1505	0.1574	0.1657	0.1778
	CLT	0.1430	0.1522	0.1591	0.1675	0.1798
	DLRA	0.1493	0.1589	0.1661	0.1749	0.1877
	CMI-3DSVD	**0.1576**	**0.1678**	**0.1754**	**0.1847**	**0.1982**
SMPI	BM3D	0.2904	0.2839	0.2760	0.2736	0.2676
	SD-BM3D	0.2968	0.2902	0.2821	0.2796	0.2735
	K-SVD	0.2931	0.2866	0.2786	0.2762	0.2702
	CLT	0.2964	0.2898	0.2817	0.2792	0.2732
	DLRA	0.3095	0.3025	0.2941	0.2916	0.2852
	CMI-3DSVD	**0.3267**	**0.3194**	**0.3105**	**0.3078**	**0.3011**
α	BM3D	0.1956	0.1941	0.1903	0.1873	0.1857
	SD-BM3D	0.2092	0.2073	0.2034	0.2012	0.1985
	K-SVD	0.1975	0.1959	0.1921	0.1890	0.1874
	CLT	0.1997	0.1981	0.1942	0.1911	0.1895
	DLRA	0.2085	0.2069	0.2028	0.1996	0.1979
	CMI-3DSVD	**0.2201**	**0.2184**	**0.2141**	**0.2107**	**0.2089**

**Table 6:** Specialist ratings for the filtered real ultrasound images.

No. of Image	BM3D	SD-BM3D	K-SVD	CLT	DLRA	CMI-3DSVD
1	5	5	5	5	5	5
2	5	5	5	4	4	5
3	5	4	5	5	3	5
4	5	5	4	4	5	5
5	4	5	4	4	5	5
6	5	5	5	5	5	4
7	4	5	5	5	5	5
8	4	5	5	4	4	5
9	5	5	5	4	4	5
10	5	4	3	5	4	5
11	4	4	4	4	5	4
12	3	4	3	3	5	5
13	4	5	4	3	4	5
14	4	5	5	4	5	4
15	3	3	4	4	5	4
16	5	3	4	5	5	5
17	3	3	5	5	4	3
18	5	4	3	4	3	5
19	3	4	5	5	4	5
20	3	5	3	4	5	5
Average	4.2	4.4	4.3	4.3	4.45	4.7

## Data Availability

The data used in this work are the simulated ultrasounds from the Field-II Ultrasound Simulation Program and the real ultrasound images from the Insana Lab: Ultrasonic Imaging (University of Illinois). They are available at https://field-ii.dk/?main.html (accessed on 16 May 2022) and http://ultrasonics.bioengineering.illinois.edu/data_patient.asp, (accessed on 16 May 2022) respectively. The data and code presented in this study are available on request to the corresponding author for academic purposes.
